# Creaming and Parking in Quasi-Marketised Welfare-to-Work Schemes: Designed Out Of or Designed In to the UK Work Programme?

**DOI:** 10.1017/S0047279414000841

**Published:** 2015-04

**Authors:** ELEANOR CARTER, ADAM WHITWORTH

**Affiliations:** * Department of Geography, University of Sheffield, UK email: ecarter1@sheffield.ac.uk; ** Department of Geography, University of Sheffield, UK email: adam.whitworth@sheffield.ac.uk

## Abstract

‘Creaming’ and ‘parking’ are endemic concerns within quasi-marketised welfare-to-work (WTW) systems internationally, and the UK's flagship Work Programme for the long-term unemployed is something of an international pioneer of WTW delivery, based on outsourcing, payment by results and provider flexibility. In the Work Programme design, providers’ incentives to ‘cream’ and ‘park’ differently positioned claimants are intended to be mitigated through the existence of nine payment groups (based on claimants' prior benefit type) into which different claimants are allocated and across which job outcome payments for providers differ. Evaluation evidence suggests however that ‘creaming’ and ‘parking’ practices remain common. This paper offers original quantitative insights into the extent of claimant variation *within* these payment groups, which, contrary to the government's intention, seem more likely to design *in* rather than design *out* ‘creaming’ and ‘parking’. In response, a statistical approach to differential payment setting is explored and is shown to be a viable and more effective way to design a set of alternative and empirically grounded payment groups, offering greater predictive power and value-for-money than is the case in the current Work Programme design.

## Quasi-marketised WTW governance: convergence in logics and tensions

The past fifteen years have seen considerable changes in how welfare-to-work (WTW) is organised and delivered across the advanced economies with a consistent trend – though with inevitable variation within it (van Berkel *et al.*, 2012) – towards ‘work first’ activation delivered by a mixture of contractualism, managerialism and marketisation (van Berkel, 2010; van Berkel *et al.*, 2012; de Graaf and Sirovatka, 2012). In the UK context, this is evident in the progression through New Deals from 1997, Employment Zones from 2001, Pathways to Work from 2007, Flexible New Deals from 2009 and, most recently, in the form of the Work Programme since 2011, each more fully and deeply embracing these principles than its predecessor. The UK has seen remarkable cross-party agreement to this approach, aided by the transfer of key policy actors such as Lord Freud from Labour to the Conservatives in 2009. Hence, the arrival of a Conservative-Liberal Democrat Coalition government in the 2010 general election after thirteen years of Labour government, and the roll-out of its flagship Work Programme WTW ‘does nothing to break out of the policy paradigm established by Labour; it simply takes it further and faster’ (Lister and Bennett, 2010: 102).

Delivery within the Work Programme takes place through contracts between the Department for Work and Pensions (DWP) and large-scale, mainly private sector, ‘Prime providers’ (Primes) who can both deliver services themselves and/or sub-contract to organisations within large and complex supply chains which sit underneath each Prime. The programme is structured geographically into eighteen large ‘regional’^1^ Contract Package Areas (CPAs) with two or three Primes in each CPA to whom claimants are randomly allocated from the public sector Jobcentre Plus if they have not found work within an initial period of Jobcentre Plus provision, usually nine to twelve months. Whilst Flexible New Deal prescribed mandatory service components, the Work Programme operates a ‘black box’ delivery model, with providers having significant flexibility over interventions to, in principle, support experimentation and harness providers' specialisation and innovation. At the heart of the Work Programme is payment-by-results for job outcomes: providers receive a small initial ‘attachment fee’ for taking a client on (though this ended in April 2014); receive a main ‘job outcome payment’ if a participant has been supported into ‘sustained paid work’ (of three months or six months duration depending on the payment group); and with further ‘sustainment payments’ available every four weeks for keeping a participant in employment (up to a maximum of twenty-four months).

In these ways, Work Programme both draws from and contributes to the shared international trend towards WTW models built around principles and practices of quasi-marketisation and new public management. However, awareness of the international evidence around such WTW systems highlights a range of by now well-known problems, tensions and risks concerning their ability to achieve what are often conflicting policy aims around efficiency, performance, innovation, personalisation and equity. Despite extensive work analysing cross-national trends towards different forms of ‘new’ WTW governance regimes since the late 1990s, there remains a distinct lack of clear evaluation evidence as to their effects (Bredgaard and Larsen, 2008; de Graaf and Sirovatka, 2012). The piecemeal evidence that does exist suggests that effects are uncertain, variable and contingent on context and implementation (van Berkel and van der Aa, 2005; Bredgaard and Larsen, 2008; de Graaf and Sirovatka, 2012). In part, this reflects the difficulty in robustly evaluating what have tended to be rapidly evolving reforms, because new ‘implementation hazards’ are thrown up as policies are implemented and as policy makers respond to problems, disappointments and shifting priorities as they emerge (Bredgaard and Larsen, 2008; van Berkel, 2010; Finn, 2011a; Finn, 2011b). The patchwork of evidence that does exist suggests that outsourced provision is not more effective than public services in achieving job outcomes, but that claimants do tend to be more satisfied with outsourced provision. The evidence also suggests that quasi-marketised WTW can – but might not (e.g., as in the Netherlands) – generate efficiency savings and that cost-cutting makes the ‘hardest to help’ claimants vulnerable, is often hampered by significant (and often significantly underestimated) ongoing pressures to re-regulate and is often achieved by providers retreating to a relatively generic and basic offer (Struyven and Steurs, 2005; Davies, 2008; Bredgaard and Larsen, 2008; Considine *et al.*, 2011; de Graaf and Sirovatka, 2012).

## ‘Creaming’ and ‘parking’ in the Work Programme: designed out or designed in?

Among these issues, of particular focus in this paper are internationally shared concerns and experiences relating to the inequitable treatment of ‘harder to help’ claimants within the context of quasi-marketised WTW programmes that operate on a payment-by-results basis – i.e., what the WTW literature refers to as problems of ‘creaming’ and ‘parking’. This is particularly true when such schemes also operate a light touch minimum standards and monitoring framework, face tight performance targets and costs pressures and/or are dominated by for-profit providers, all of which apply in the case of the UK Work Programme. In the context of WTW interventions, ‘creaming’ refers to provider behaviour that prioritises attention for unemployed claimants with fewer barriers to work and who are therefore felt to be easier, cheaper and also more likely to move into paid work and release outcome payments. In contrast, ‘parking’ refers to provider behaviour that deliberately neglects giving time, energy or resources to unemployed claimants with more substantial barriers to work, given that such claimants are considered to be relatively unlikely to move into paid work and/or to require considerable, and usually expensive, employment support to make a move into paid work likely (and hence an outcome payment). The practice of creaming and parking clearly cuts against the explicit policy intention to ‘narrow the performance gap between easier and harder to help claimants’ (NAO, 2012: 5), but the financial and psychological implications of the practice are also of concern. Creaming and parking effectively translate to overpayment by government for any given employment outcome secured, and for individual programme participants who are denied meaningful support there are serious ramifications since unemployment, when coupled with lack of progression and ability to control the future life course, is associated with reduced mental well-being (Fryer, 1986). Given that providers receive payments based on job outcomes, there is a core tension around how best to deal with the inevitable variation in the likelihood of different claimants' successfully moving into paid work (and hence releasing outcome payments). It is important therefore for programme design to ensure that the incentives of the economically rational provider to cream or park claimants, depending on type, is mitigated such that meaningful and equitable employment support is provided for all, irrespective of the level, complexity and cost of their support needs.

Mitigating such risks is a perennial design challenge within quasi-marketised WTW schemes using payment-by-results, and one that international experiences show to be difficult to resolve. This might be expected to be particularly true during periods of sustained economic difficulty, when jobs are scarce, when the economic context is more challenging than it was at the point at which contracts and performance targets were drawn up, when funding is largely or wholly based on successful job outcomes such that up-front funding for more costly interventions is harder to find and when providers are explicitly for-profit. These are all situations applicable to the Work Programme since its inception, though Rees *et al.* (2013) find that sector makes less difference than might perhaps be expected given the sharp financial pressures experienced by all providers within the scheme to date. More broadly, it should be noted that failings in meeting the support needs of harder-to-help claimants are not exclusive to outsourced provision, and, in part, reflect the reality that providers – of whatever sector – simply may not have the scope or resources to affect certain characteristics of claimants (e.g., mental or physical health conditions) or their local areas (e.g., a chronic lack of suitable jobs locally) that are relevant to their (un)employment.

Despite inevitable variation in results, the international evidence is, on balance, clear about the risk and existence of creaming and parking in outsourced, payment-by-results WTW models (Struyven and Steurs, 2005; van Berkel and van der Aa, 2005; Bredgaard and Larsen, 2008; van Berkel *et al.*, 2012; de Graaf and Sirovatka, 2012). The international evidence is equally clear about the central, dynamic, enormously challenging and largely trial-and-error process of programme design attempts to effectively guard against practices of creaming and parking (Struyven and Steurs, 2005; Finn, 2009; Finn, 2011a; Finn, 2012). A range of different design approaches have been proposed and pursued internationally to mitigate the risks around creaming and parking, and these can be grouped into six main strands that can be employed either in isolation or in combination:

• **Accelerator/progressive pricing systems** in which payments for job outcomes increase in size as providers reach further into their caseloads, in principle incentivising them to support claimants with more complex needs who are assumed not to be amongst the early movers into paid work.
• **Different programmes or streams for different claimant groups** so that differently tailored support, performance criteria and outcome payments can be more easily and more explicitly operated for different types of claimants.
• **Differential pricing for individual participants within a holistic programme** so that providers are incentivised to work meaningfully with all unemployed claimants, irrelevant of the level of their support needs/costs because the outcome payments attached to claimants increase in line with these support needs/costs.
• **Milestone payments** can be used to incentivise providers to focus on all claimants despite their different places and trajectories on their employment journey (e.g., for moving claimants closer to work even if they do not move into work, for transitions from unemployment into work, or to reward job retention or progression for those in work).
• **Claimant leverage** can be used systemically to seek to drive up provider performance by creating effective channels for unemployed participants to exercise voice (e.g., effective complaints processes and/or customer surveys) and exit (e.g., participant agency around choosing or switching providers or interventions).
• **‘Star ratings’**, as used in the Australian WTW model, where provider performance is rated based on their outcome performance after having controlled for the characteristics of claimants and their local areas, hence seeking to create a fairer comparison between providers with more and less difficult caseloads.

Of these potential policy responses, the Work Programme seeks to mitigate creaming and parking through minimum service guarantees from Prime providers and, particularly, via the use of nine separate payment groups with differential payments across these groups. Of these two mechanisms, weaknesses in the substance, consistency and, in some cases, even the possible enforceability of providers’ minimum service guarantees render these a far less useful and reliable protection than they could be (Finn, 2012). Moreover, the inherent vulnerability of Work Programme providers’ minimum service guarantees combines with highly imperfect participant awareness both of these guarantees and of the complaints procedures – particularly the existence of the Independent Case Examiner – through which claimants might complain and seek improved service. In practice, therefore, there is a reliance within the Work Programme on the effectiveness of the payment groups and the differential pricing structure to defend against creaming and parking (Finn, 2011b: 2).

As shown in Table 1, Work Programme participants are grouped into nine payment groups across which maximum possible payment levels for providers vary. This segmentation operates, in the DWP's view, according to some notion of the average difficulty of securing transitions to employment for different claimants. As noted above, providers can receive three types of payments – an initial attachment fee (this disappeared in April 2014), a job outcome payment when a claimant moves into ‘sustained employment’ (work of three or six months depending on the payment group) and further monthly sustainment payments when a claimant remains in paid work. This payment model is significantly predicated on realising successful job outcomes such that up-front funding to providers for interventions is tight while pressures to deliver job outcomes are intense, due both to the reliance on outcome payments but also to the permanent threat of contract termination for providers deemed by the DWP to be underperforming.^2^

**TABLE 1.** Work Programme payment groups and payment levels

ClaimantGroupnumber	CustomerGroup	Time ofreferral	Basis forreferral	Max Payment Year 1 starters (£)	% of Work Programme population
1	JSA 18–24	From 9 months JSA	Mandatory	3,810	20.09
2	JSA 25+	From 12 months JSA	Mandatory	4,395	44.94
3	JSA Early Access	From 3 months JSA	Mandatory or voluntary depending	6,600	24.65
4	JSA ex-IB	From 3 months JSA	Mandatory	6,600	0.71
5	ESA volunteers	At any time from WCA	Voluntary	3,700	1.76
6	New ESA claimants	Mandatory when expected to be fit for work in 3–6 months; voluntary from WCA	Mandatory or voluntary depending	6,500	5.92
7	ESA ex-IB	Mandatory when expected to be fit for work in 3–6 months; voluntary from WCA	Mandatory or voluntary depending	13,720	1.03
8	IB/IS (England only)	At any time	Voluntary	3,285	0.23
9	JSA prison leavers	On release	Mandatory	6,600	0.67

On first impressions, the complex system of payment groups, differential pricing and milestone payments appears a reasonably robust primary defence against provider incentives to cream and park claimants. Such a view would certainly tally with DWP's own confidence that it will ‘ensure that providers have strong incentives to help all of their customers’, and will close the performance gap between the easiest- and hardest-to-help (DWP, 2012: 6). However, the Work Programme payment groups are defined relatively crudely on the basis simply of the type of benefit being claimed at the point of referral. This is in contrast to other systems internationally – most notably the Australian Jobseeker Classification Instrument (JSCI) – that combine complex differential pricing systems with significantly more subtle profiling instruments. One parking risk is that payments simply may not be high enough to realistically cover the intervention costs of the hardest to help, and recent evidence suggests that this may indeed be an important ongoing problem in the scheme (WPSC, 2013; Riley *et al.*, 2014). Of central concern to the present paper, however, is the widely discussed but as yet unquantified risk that the Work Programme's broad payment groups may actually offer up incentives for providers to cream and park given that, within such broad and heterogeneous payment groups, all claimants attract the same job outcome payment, but there is known to be substantial variation in support needs/costs, employment likelihood and risk for providers across those claimants (WPSC, 2011, 2013).

Given that these issues have been widely experienced and discussed internationally, one may ask how Work Programme's rather simplistic payment groups came to be, with the DWP offering differential pricing across payment groups as the primary systemic defence against creaming and parking incentives within the programme. Chris Grayling, then Minister for Employment within the DWP explained, ‘we needed to find something that was simple to administer that was likely to be reasonably reflective’ and that ‘was simple, easy to understand, where there was no scope for debate and discussion . . . There will be variations within each group, that is inevitable but we think as a broad average it gives the providers a sensible basis to work with’ (Grayling in WPSC, 2011: Ev40; 28). Robert Devereux, Permanent Secretary of the DWP, mirrors this sentiment that the Work Programme payment groups and differential payments system ‘begins to move us towards trying to reflect some of the average difficulty [of moving claimants into sustained employment]. Everything we have done here takes us really a long way forward compared with where we were’ (PAC, 2012: 26).

It is undoubtedly true that the Work Programme's differential payment design is administratively simple, easy to explain and understand, and more nuanced then previous schemes. Yet, it has also been repeatedly pointed out that the current segmentation based on benefit type is an overly – and, in our view, unduly – crude basis on which to proxy distance to labour market and, as a consequence, on which to calibrate groups and differential payment levels (Lane *et al.*, 2013; WPSC, 2011; WPSC, 2013). Rather, the more appropriate question is not, as Robert Devereux suggests, whether the current Work Programme design is more complex than previous designs in a relative sense – it is (WPSC, 2013: 33) – but, rather, whether or not it is adequate and effective in an absolute sense in mitigating provider incentives to cream and park and, in so doing, to deliver effective support for all claimants as well as value-for-money for the taxpayer. Here, unfortunately, there is growing and consistent evidence from within DWP's official evaluation (Newton *et al.*, 2012; Lane *et al.*, 2013), from select committees (PAC, 2012; PAC, 2013; WPSC, 2011; WPSC, 2013) and from academic research (Rees *et al.*, 2013; Rees *et al.*, 2014) that this is not the case. Indeed, the first phase of the DWP commissioned qualitative evaluation of the Work Programme is as clear as any government commissioned evaluation is ever likely to be: ‘the available evidence to date suggests that providers are engaging in creaming and parking, despite the differential payments regime’ (Newton *et al.*, 2012: 124). One further telling piece of evidence is that Prime providers are using in-house profiling to develop their own claimant streams rather than adopting DWP's claimant groups as the structure for their activities, suggesting that the current payment-group-based differentiated payments do not correspond to providers’ views of claimants’ support needs and distance to labour market (Newton *et al.*, 2012; Lane *et al.*, 2013). Indeed, one provider suggested that the RAG rating profiling system^3^^3^ used by some Primes and their end-to-end providers to ‘triage’ their caseloads was in effect a mechanism for creaming and parking:

So from day one you're categorised and if you're a green customer you've got an anticipated job start date, you've got an action plan to work towards that, and you have to be seen so that is once or twice a week. So you're pushed. If you're amber your job start date is obviously further away, and it's the expectation that you'll have activity at least once a fortnight. If you're red it could be a phone call once a month. So people are not using the word parking because it's politically incorrect, but it's happening [Tier 2 provider]. (Rees et al., 2014)

The focus of this paper is to offer an original, programme-wide quantitative assessment of the extent of claimant variation within payment groups and, as a result, the extent to which the Work Programme's current payment-group based differential payment system may be designing in rather than, as intended, designing out provider incentives to cream and park claimants. Following on from this, the paper explores the potential for greater use of statistical profiling techniques to better calibrate Work Programme's differential payment levels with claimants’ distance from the labour market as one element towards creating an improved future Work Programme; a timely empirical contribution given recent requests from the DWP Select Committee for fuller consideration of this approach (WPSC, 2013).

## Data and methods

To explore these issues, the analyses below make use of the first two waves of the special licensed geocoded version of Understanding Society, the UK's largest household survey, to look at whether the current policy design links in to evidence of differing likelihoods of sustained employment transitions amongst a cohort of individuals identified to fit within matched pseudo-payment groups.

Some form of profiling within WTW systems is almost inevitable and is, in different countries and for different schemes, usually based on simple administrative eligibility criteria, such as in the Work Programme (e.g., benefit type), advisor discretion or statistical modelling. Statistical approaches within WTW are considerably more developed in the US, but improvements in data quality and availability in the UK over the past decade have been shown to enable statistical profiling to an acceptable degree of accuracy, even if there will always be error, and evaluations of ‘acceptable accuracy’ will always to some extent be subjective (Hasluck, 2004; Bryson and Kasparova, 2003; Matty, 2013).

Work Programme pseudo-payment groups were identified from Wave 1 of the Understanding Society survey (University of Essex. 2009–2011) in accordance with DWP's service provider guidance^4^ (summarised in Table 1 above). Due to the small cohort size of some Work Programme payment groups, the Understanding Society data, despite their size, do not allow all nine claimant groups, or all parts of claimant groups, to be identified. However, four key payment groups – Jobseeker's Allowance (JSA)^5^ claimants aged eighteen to twenty-four, JSA claimants aged twenty-five plus, JSA Early Access, and ESA^6^ Work Related Activity Group (WRAG) – were included and together these payment groups account for around 90 per cent of the Work Programme referrals to date. In total, 933 such pseudo Work Programme individuals were identified in Wave 1, with 57 per cent of these cases in the JSA twenty-five plus payment group.

The identification of individuals in the JSA eighteen to twenty-four and the JSA twenty-five plus payment groups is relatively straightforward. The JSA Early Access group contains three separate types of claimants: mandatory entry of young adults not in education, employment or training (NEETs); mandatory entry of ‘JSA repeaters’;^7^ and voluntary early entry of a series of pre-identified ‘vulnerable’ JSA claimants (e.g., homeless, carers). It is possible in the Understanding Society survey to identify eligible NEET participants, but not voluntary ‘vulnerable group’ participants. JSA repeaters can be identified through the employment history module, but only for the one-quarter of the main sample of whom this is asked. Hence, our JSA Early Access cohort omits voluntary ‘vulnerable’ participants and under represents JSA repeaters.

New ESA claimants can be either mandatory^8^ or voluntary^9^ participants depending upon their circumstances. It is not possible to estimate voluntary participation, and our New ESA payment group is comprised therefore only of mandatory ESA WRAG participants. These individuals were identified according to the severity of their health condition where we mimicked as closely as possible the questions within the Work Capability Assessment (WCA) from the various health-related survey questions within Understanding Society. DWP (2013:10–11) reports that 56 per cent of the ESA decisions resulting from the WCA, place individuals within the WRAG group rather than the Support Group and the least severely disabled 56 per cent of these ESA claimants in the survey are therefore classified by us as ESA WRAG and are mandated into this payment group.

Having identified the pseudo-payment groups, employment outcomes over approximately the following twelve months were tracked. Of the 933 individuals originally identified in Wave 1, 528 individuals remain across our four pseudo-payment groups once survey attrition, missing employment data and residency beyond Great Britain^10^ are accounted for. Of these 528 individuals, 59 per cent fall in the JSA twenty-five plus payment group and roughly 14 per cent in the remaining three payment groups. The key outcome variable is transition into ‘sustained employment’ as it is this that triggers the Work Programme job outcome payment. Transitions to ‘sustained employment’ are defined as six months of cumulative employment for the JSA eighteen to twenty-four and JSA twenty-five plus groups and as three months of cumulative employment for the JSA Early Access and New ESA groups. Whilst providers can also receive sustainment payments for helping claimants to remain in paid employment, the job outcome payment is a key outcome measure of the programme and makes for a sensible basis on which to evaluate the adequacy of the payment group structure. Around one-third of our cases in each payment group experience a transition into employment over this period, although only 18 per cent of cases show a transition into the ‘sustained employment’ that is the focus in the modelling below.

**Figure 1.**
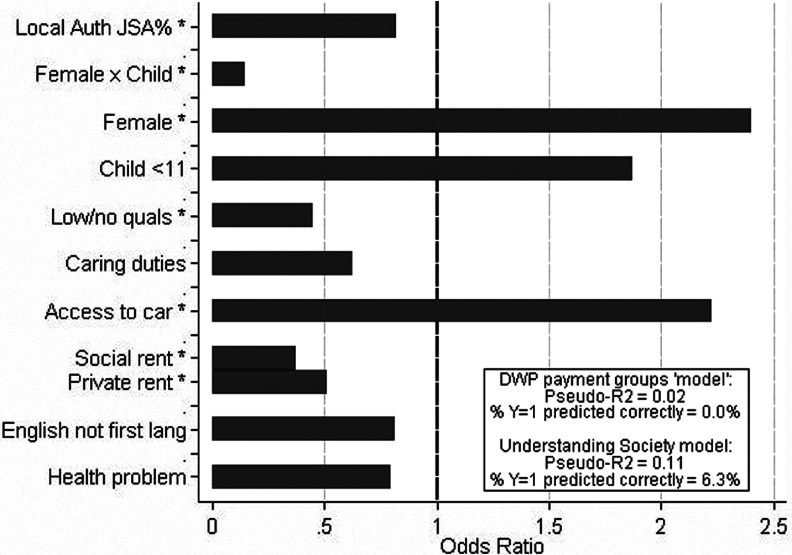
Explaining transitions into sustained employment

Binary logistic regression models are used to estimate these claimants’ predicted probability of transition into sustained work, given their characteristics, and Understanding Society offers a rich set of explanatory variables for potential inclusion. Model testing identified the set of variables shown in Figure 1 as the most parsimonious^11^and variables are retained either when they are statistically significant or when they show reasonable effect sizes and their lack of statistical significance could plausibly be due to small sub-group sample sizes. This is in recognition of the fact that our sample size is limited and that our desire is to focus on messages and effect sizes at least as much as simple statistical significance (Ziliak and McCloskey, 2008). It also reflects our desire to present exploratory analyses and ideas around the potential role of statistical profiling as one potential element within a reformed Work Programme. Follow-on research with larger and richer administrative and/or Prime provider data that are not currently available in the public domain could be incorporated as a next step.

## Identifying and mitigating provider incentives to cream and park through enhanced profiling

Figure 1 summarises the odds ratios from the final regression model with asterisks used to highlight effects that are statistically significant at the 5 per cent level. The substantive direction and size of these effects is as expected, though the restricted sample sizes for some sub-groups limits our ability to detect statistical significance for some findings even when these effects are relatively large in size (e.g., experiencing health problems, English not first language).

Our focus is less on individual effects within the model and instead offers model fit as a way of evaluating the current Work Programme design, as well as highlighting the potential of statistical approaches to drive subtler profiling and payment setting to better protect against creaming and parking. There is no agreement in the literature as to how best to evaluate the model fit of binary logit models such as these and a box to the lower-right of Figure 1 shows two commonly used model fit statistics – pseudo-R2 and predicted groups (i.e., whether an individual's transition to employment is successfully predicted). As discussed below, although these measures are problematic, when modelling rare events such as these they are presented here in order to get a feel for how our statistical model from Understanding Society performs compared with the current DWP Work Programme design. The lower section relates to the Understanding Society regression and shows a pseudo-R2 of 11 per cent, with 6.3 per cent of the actual transitions into sustained employment being correctly predicted. By way of comparison, the upper section presents equivalent model fit statistics for a separate regression (not shown) relating to the current DWP Work Programme ‘model’ in which only payment groups are used as the predictor of transitions into sustained work. This DWP model offers virtually no explanatory power on these measures and this raises serious questions about the evidence for, and logic of, the current Work Programme payments structure.

More importantly, in terms of this paper's argument, this comparison highlights the relative superiority of a statistical approach to profiling and policy structuring. However, one technical challenge is that neither model fit is well suited to assess the power and potential of a statistical approach, as a viable profiling strategy in this case, given that the sustained employment transition outcome variable being modelled is relatively rare. Pseudo-R2 statistics from logit models lack the transparent, agreed upon, and conceptually sound foundations of their better-known OLS counterparts and, consequently, are often not reported. As a result, a variety of such statistics exist, all with health warnings for users and all tending to produce relatively low values when seeking to model rare events. The principles of the predicted groups measure is more helpful given that it is this, after all, that Prime providers will be paid for – can we use statistical models to differentiate between different types of unemployed claimants in terms of their likelihoods of moving into sustained work? The measure in its current form, however, is not well suited to this task, given that the threshold value to allocate cases to groups is predicted probabilities above 0.5 (predicted a sustained work transition) or below 0.5 (not predicted a sustained work transition). Although arbitrary, this default is usually unproblematic. When outcomes are rare, however, a threshold value of 0.5 for the predicted probabilities is unrealistic as a basis to allocate cases given that, almost by definition, these long-term unemployed individuals are not (in a probabilistic sense) highly likely to enter sustained employment. Hence, and as shown in the left pane of Figure 3, few cases gain predicted probabilities above 0.5, leaving an inevitably low percentage of the cases that do actually move into sustained work with correct predictions. The default predicted group measure is not a helpful one in these circumstances. Moreover, the policy imperative for the Work Programme in terms of profiling and differential payments is the particular need to differentiate between the *relative* likelihood of unemployed individuals moving into sustained employment far more than it is to fully account for those employment transitions; the two are related, but they are not identical.

**Figure 2.**
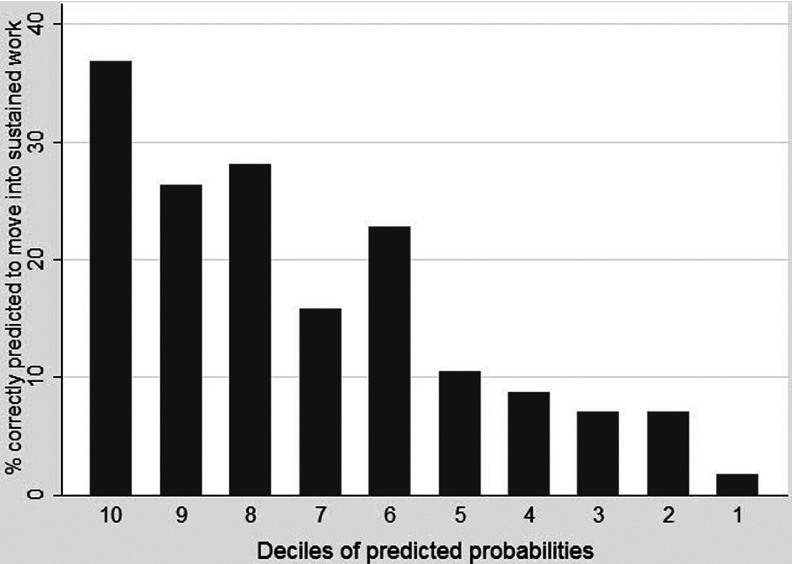
Claimant segmentation and predictive accuracy

**Figure 3.**
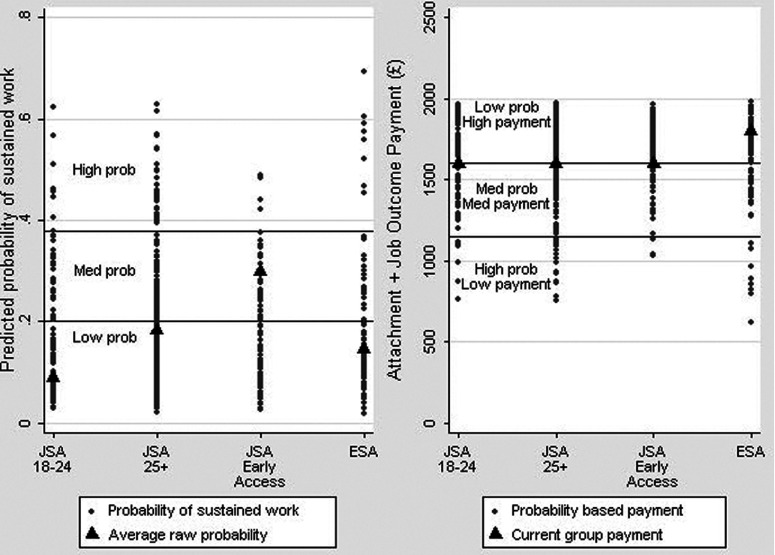
From predicted probabilities to an improved differential payments system

Recent work by the DWP to estimate the likelihood that the unemployed will become long-term unemployed (Matty, 2013), the inverse in many ways to our focus, faces a similar problem and a similar technical challenge. It suggests segmentation analysis as a way forward as both a more conceptually and technically meaningful approach to the evaluation of model power in these circumstances. In this segmentation approach shown in Figure 2, claimants’ predicted likelihoods of moving into sustained employment are broken into ten equally sized deciles with the 10 per cent of cases with the highest probabilities in the far left bar of Figure 2 through to the 10 per cent of cases with the lowest probabilities in the far right bar of Figure 2. For each segmented group the vertical axis shows the percentage of each group that did actually move successfully into sustained work in these survey data. For example, the far left bar shows that, of the 10 per cent of cases predicted most likely to move into sustained employment, around 37 per cent actually did so. In general, and crucially, the model correctly orders claimants’ relative likelihoods of moving into sustained work as shown by the gradual decline from left to right, such that at the far right of Figure 2 less than 2 per cent of the least likely claimants to move into sustained work actually do so. Due to the relatively small sample size of just over fifty cases in each decile in Figure 2, there is some inevitable crudeness in these data, shown most clearly by the slight jump in decile 6, and greater subtlety and smoothness would be expected with larger datasets. Even with these data, however, Figure 2 highlights that it is possible to use the relative positioning of the predicted probabilities to fulfil the key policy task required for meaningfully differentiating *between* the long-term unemployed in terms of their likelihood of moving into paid work.

The statistical model is not perfectly predictive, naturally, but it is superior to the current DWP model in an absolute sense (see Figure 1) and, most importantly in terms of its policy function, it is able to accurately and sensibly differentiate between different types of unemployed claimants (see Figure 2). In our view, this offers positive proof of concept for the potential of statistical profiling as a viable route towards subtler statistically based approaches to differential payment structures. The future availability of larger, richer administrative and/or Prime provider data could offer real traction for improving such approaches further. At the same time, statistical profiling approaches should not be seen as a ‘magic bullet’ for solving the challenge of creaming and parking within the Work Programme, but, instead, as one necessary element in a broader evolution towards a redesigned Work Programme offering better calibrated incentives for providers and improved value-for-money for the taxpayer.

How, though, might a statistical approach to profiling and payment structures potentially be operationalised in a redesigned future Work Programme? Ignoring for a moment the horizontal lines and labelling in Figure 3, the grey dots in the left pane of Figure 3 plot the individual predicted probabilities of moving into sustained work for our Understanding Society sample. Although the logic of our approach steps beyond the existing payment groups structure, quantifying and visualising this variation within the existing payment groups is a powerful visual insight into the extent to which highly cost-pressured, mainly for-profit Prime providers are currently facing considerable incentives to profile, cream and park claimants in the Work Programme. Specifically, if one assumes economic rationality on the part of providers then, given that all claimants within each payment group share the same payment level, those individuals with the lowest predicted probabilities of moving into sustained work seem to be at considerable risk of being parked, while those individuals with the highest predicted probabilities seem liable to be creamed. Indeed, given the likelihood of increased intervention costs for harder-to-place claimants, the incentives to park and cream are stronger than these probabilities suggest. Equally, individuals in different payment groups, but with equivalent predicted probabilities of moving into sustained work, might be expected to be treated differently by Primes where payment levels differ substantially between those payment groups. The black triangles in this left pane of Figure 3 reflect the payment group's *average* probability of moving into sustained work – in other words DWP's basis for the current payment groups and pricing structure. Figure 3 makes clear that this, in Chris Grayling's words, ‘broad average’ (Grayling in WPSC, 2011: 28) is precisely that, and is a poor basis for profiling and price setting given that virtually all of the variation shown is *within* rather than *between* payment groups.

One obvious way to seek to better protect against creaming and parking given these two conflicting features of the current Work Programme system – large individual variation within payment groups combined with fixed payment levels for those payment groups – is to calibrate payment levels more closely with modelled likelihoods of moving into sustained employment. Although visualised here within payment groups for ease of explanation, there is no need to retain the existing payment groups within any such redesign, and, indeed, it may well be more logical to move beyond them as the Work Programme evolves.

The right pane of Figure 3 shows two potential redesigns. Firstly, one might in principle think about individualised payment levels, though this does not represent the preferred way forward in our view. The black triangles in this right pane of Figure 3 reflect the payment levels that providers currently receive once an individual in each payment group moves into sustained employment (i.e., the attachment fee plus job outcomes payment). The vertical spread of grey markers show one potential payment redesign: individualised payments based on each individual's predicted probability of moving into sustained paid work and calculated as an inverse share of the current budgetary pot (i.e., this proposal is cost-neutral). As a result, providers are given strengthened financial incentives to work with individuals with lower predicted probabilities who are farther from the labour market (markers lower down in the left pane) and who are most at risk of being parked in the current framework. The flip-side of this analysis, of course, is the recognition that the DWP must currently be systematically overpaying providers for many job outcomes *if* it is accepted that parking is taking place *and* that payment levels reflect average payment group likelihoods of moving into sustained work. Hence, the need for an improved payment structure relates importantly also to efficiency (taxpayer value-for-money) as well as to equity (creaming and parking).

In our view, however, despite the improvement to claimant segmentation that statistical profiling can offer, it may well be sensible to remain realistic about the error that will always occur at the individual level within such approaches. Hence, a sensible route forward is, in our view, to think instead more about creating alternative, empirically based and probabilistically grounded payment groups rather than seeking fully individualised payment levels. The horizontal lines and plot labelling in Figure 3 show how such an approach might work. In this example, Figure 3 identifies three new groups of claimants with low probabilities/high payments, medium probabilities/medium payments and high probabilities/low payments, respectively. Relating this back to Figure 2 above, it may be for example that the top three deciles with the highest predicted probabilities form one group (with lower payments), the central three deciles form a second group (with medium payments) and the bottom four deciles with the lowest predicted probabilities form a third group (with higher payments), or some such equivalent. This is the essence of the Job Services Australia model that has been in place since 2009 in Australia and in which claimants are placed into one of four differently paying streams according to their distance from the labour market as based on a detailed statistical profiling exercise known as the Job Seeker Classification Instrument.

## Discussion

Outsourced delivery of WTW support, based on payment-by-results, is becoming an increasingly widespread policy framework across advanced economies, and the UK Work Programme is in many ways at the vanguard of this trend. A perennial problem within such frameworks is the difficulty in incentivising what are often profit-seeking providers to support all claimants effectively, despite their differing support costs and their differing likelihoods of realising outcome payments. Within the Work Programme, it is differential pricing across administratively defined payment groups that seeks to calibrate provider incentives across the whole range of claimants and, in so doing, to mitigate risks of creaming and parking for claimants and to realise value-for-money for taxpayers. The evaluation literature to date, however, suggests that the current Work Programme design is not achieving these aims and that creaming and parking do seem to be taking place. At the heart of this problem lie the overly simplistic current payment groups, and a pricing mechanism that hides significant within-group variation and that, as a consequence, is a poor counter to providers’ economically rational incentives to cream and park differently positioned claimants.

The modelling work presented above offers original and timely programme-wide quantitative evidence on this debate for the first time. It highlights that the current Work Programme payment groups offer virtually no explanatory power given that the vast majority of the variation in predicted probabilities is *within* rather than *between* payment groups. The incentives for creaming and parking within this context are considerable and there is a real risk that the current payment groups unintentionally design such practices *in* rather than *out*. In response, the analyses highlight that there is viable potential in the UK context for a statistical approach to profiling and improved payment calibration in order to create alternative, empirically grounded and probabilistically rooted payment groups as in the Australian model. The result of doing so would be to better calibrate Work Programme price setting, more effectively design out creaming and parking and better protect taxpayer value-for-money than is the case currently. Given that a switch towards some form of statistical profiling approach is relatively simple, and inexpensive to implement and explain, it is unclear why it would not be seriously considered given its empirical advantages to the current Work Programme design. These analyses in our view offer positive proof of concept, and further work with larger and richer administrative and/or Prime provider data is now needed to progress this work towards use in the field.

At the same time, statistical approaches to improved profiling, segmentation and payment calibration clearly should not be expected to offer a magic bullet to these problems and, indeed, they come with problems of their own (Struyven and Steurs, 2005: 221; OECD, 2012: 110–112). The Australian system, for example, towards which these analyses point, has undergone successive waves of policy reform as it has sought to respond variously to problems experienced around parking, employer engagement, participant group misallocation and ‘gaming’ by providers (Finn, 2011b). As outlined above, a range of approaches can be taken to achieve the multiple aims of quasi-marketised WTW schemes, such as the Work Programme, and many of these approaches should be considered as necessary complements to one another rather than as alternative options. While statistical approaches offer clear advantages to the current Work Programme payment structure, other complementary design changes are also likely to be necessary if both the hardest-to-help claimants and value-for-money for the taxpayer are to be supported effectively. Obvious areas to enhance within the Work Programme include tightening up on the existing variable and often unduly vague minimum service guarantees, greater attention to, and usage of, claimant experiences and claimant choice. Key questions also remain around the adequacy of current resources to cover the intervention costs of those with complex support needs, whether the current balance between up-front and outcome-based payments is the right one and whether intermediate outcome payments might also be used more creatively. One example of the latter might be to incentivise providers to reduce the distance to labour market for those claimants with the most substantial barriers where transitions to work itself simply may not be realistic in the short-term.

As outlined above, the trends towards these new governance regimes within WTW delivery run considerably ahead of the evidence of their positive impacts yet there seems little likelihood of anything but a general continuation of these shifts. If that is the case, and we are to remain within the logic and practice of that paradigm, then the least that we should do is to remain flexible to reshaping those policies as evidence emerges regarding their weaknesses and limitations. The present analyses offer a starting point for one aspect of these discussions. It is hoped that they will stimulate serious academic and policy debate in the UK about the evident need for Work Programme evolution around its currently problematic approach to profiling and differential pricing that seems to be designing in, rather than designing out, provider incentives to cream and park.

## Notes

1 The North East and Scotland are examples of the scale of CPAs.

2 In March 2014, Newcastle College Group became the first Prime provider to have one of its contracts ended, despite the flawed design of the minimum performance levels and despite NCG technically not having breached those minimum performance levels (NAO, 2014).

3 RAG rating uses a traffic light system to group individuals either as red (hard to move into work), amber (moderately difficult) or green (easy to move into work).

4 Department for Work and Pensions (2014).

5 JSA refers to Jobseeker's Allowance, the main out-of-work benefit for unemployed individuals without a certified health condition in the UK.

6 ESA refers to Employment and Support Allowance, the main out-of-work benefit for the unemployed with a certified health condition in the UK (although ESA can also be claimed by those in work).

7 Those who have received JSA in twenty-two of the past twenty-four months.

8 ESA Work Related Activity Group (WRAG).

9 ESA claimants with child under five and either three to six month prognosis or a full-time carer.

10 Some survey cases relate to Northern Ireland but Work Programme operates only in Great Britain.

11 Also tested but not retained in final model were: age, ethnicity, household type, vocational qualifications, number of children, work experience, born in UK, urban/rural, uses public transport rather than car.
